# To the Readers of the Journal of Comparative Medicine and Surgery

**Published:** 1889-10

**Authors:** W. A. Conklin


### EDITORIAL DEPARTMENT.

To the Readers of The Journal of Comparative Medicine and Surgery.—Commencing January 1, 1890, this Journal will be published monthly instead of quarterly, as heretofore. It will be under the chief editorial charge of Dr. R. S. Huidekoper. Articles by the best writers will still be found in its pages, among them a monograph on Odontoms in man and lower animals by J. Bland Sutton, F. R. C. S., as well as selections from the European veterinary journals.

In making this change we have been actuated by the desire to place before our readers at more frequently recurrent periods the results of the most recent investigations in veterinary science, as well as the practical work which is of so much interest to the profession.

There is no doubt that in its new career under Dr. Huidekoper's management the Journal will be eminently successful.

We predict for it a magnificent future, and feel confident that the undertaking will inaugurate a new era in the development of veterinary medicine.

W. A. Conklin.

